# Deciphering the Role of 3D Genome Organization in Breast Cancer Susceptibility

**DOI:** 10.3389/fgene.2021.788318

**Published:** 2022-01-11

**Authors:** Brittany Baur, Da-Inn Lee, Jill Haag, Deborah Chasman, Michael Gould, Sushmita Roy

**Affiliations:** ^1^ Wisconsin Institute for Discovery, University of Wisconsin-Madison, Madison, WI, United States; ^2^ McArdle Laboratory for Cancer Research, University of Wisconsin-Madison, Madison, WI, United States; ^3^ Department of Biostatistics and Medical Informatics, University of Wisconsin-Madison, Madison, WI, United States

**Keywords:** window of susceptibility, breast cancer, 3D genome organization, gene regulation, matrix factorization

## Abstract

Cancer risk by environmental exposure is modulated by an individual’s genetics and age at exposure. This age-specific period of susceptibility is referred to as the “Window of Susceptibility” (WOS). Rats have a similar WOS for developing breast cancer. A previous study in rat identified an age-specific long-range regulatory interaction for the cancer gene, *Pappa*, that is associated with breast cancer susceptibility. However, the global role of three-dimensional genome organization and downstream gene expression programs in the WOS is not known. Therefore, we generated Hi-C and RNA-seq data in rat mammary epithelial cells within and outside the WOS. To systematically identify higher-order changes in 3D genome organization, we developed NE-MVNMF that combines network enhancement followed by multitask non-negative matrix factorization. We examined three-dimensional genome organization dynamics at the level of individual loops as well as higher-order domains. Differential chromatin interactions tend to be associated with differentially up-regulated genes with the WOS and recapitulate several human SNP-gene interactions associated with breast cancer susceptibility. Our approach identified genomic blocks of regions with greater overall differences in contact count between the two time points when the cluster assignments change and identified genes and pathways implicated in early carcinogenesis and cancer treatment. Our results suggest that WOS-specific changes in 3D genome organization are linked to transcriptional changes that may influence susceptibility to breast cancer.

## Introduction

A major goal of breast cancer research is to prevent cancer. Breast cancer susceptibility by environmental exposure is modulated by an individual’s genetics and age at exposure. For example, environmental or diagnostic radiation exposure poses a high risk to women in early childhood to young adult stage and is significantly reduced starting in the mid-30s (Terry et al., 2019). This age-specific period of high susceptibility is referred to as the window of susceptibility (WOS). Large scale consortia efforts in breast cancer research have significantly advanced our ability to identify genomic loci and molecular pathways that contribute to breast cancer susceptibility (Koboldt et al., 2012; Welter et al., 2014). However, the gene regulatory mechanisms in the WOS remain poorly characterized.

Three-dimensional (3D) organization of the genome, which defines how the DNA is packaged inside the nucleus has emerged as a major component of the gene regulation machinery in mammalian genomes (Bonev and Cavalli, 2016). Three-dimensional genome organization enables long-range interactions between distal regulatory sequences, such as enhancers, and target gene(s) through chromosome looping that brings the regulatory element in close spatial proximity to the target gene. In addition to looping patterns, the chromatin is organized into high-order structural units such as topologically associating domains (TADs) within the cell (Dixon et al., 2012; Hou et al., 2012; Sexton et al., 2012). TADs refer to groups or clusters of genomic regions that preferentially interact among themselves (Bonev and Cavalli, 2016; Rowley and Corces, 2018).

Changes in 3D genome organization, both at the loop and the TAD levels, have been associated with developmental and disease processes (Chakraborty and Ay, 2018; Zheng and Xie, 2019). In particular, genome-wide chromatin looping has been shown to occur in a stage-related manner in the developing limb (Andrey et al., 2017) and in blood cell differentiation (Javierre et al., 2016). TAD-level changes have been associated with timepoint-specific regulatory interactions during differentiation and development (Hug and Vaquerizas, 2018; Paulsen et al., 2019; Zheng and Xie, 2019). Disruptions in TADs have also been associated with numerous diseases including cancer. Delayed replication of large genes near TAD boundaries underlies common fragile sites, hotspots of chromosome instability in cancer (Sarni et al., 2020). Furthermore, disruptions to the TAD-level interaction patterns have been implicated in oncogenesis (Hnisz et al., 2016; Taberlay et al., 2016; Rhie et al., 2019).

Genome architecture has been implicated in cancer susceptibility due to environmental factors (Henning et al., 2016; García-Nieto et al., 2017). For example, lamina-associated heterochromatin at the nuclear periphery is more susceptible to ultraviolet radiation, an environmental carcinogen that causes skin cancer, compared to accessible euchromatin (García-Nieto et al., 2017). At the individual loop level, a 8.5 kb regulatory element, called the temporal control element (TCE) was shown to interact with the *Pappa* gene via long-range chromatin looping of 517 kb (Henning et al., 2016) in both breast cancer resistant rats and susceptible rats. This element lies within the 170 kb *mammary cancer susceptibility (Mcs5c)* locus, a gene desert on rat chromosome 5 which is conserved in the human genome. Furthermore, this interaction was dependent upon the age of the rat, being stronger in young rats (in WOS) versus old rats. Correspondingly, *Pappa* expression was increased in susceptible rats compared to resistant rats within WOS and there was no difference between the two alleles in the adult phase. The *Pappa* gene is a breast cancer-associated gene, which positively regulates the IGF signaling pathway and is important for normal mammary gland development. This is the first validated example of WOS-specific chromatin looping. However, the contribution of the loops and TADs on a genome-wide scale to breast cancer risk from enviromental factors in the window of susceptibility is poorly understood.

To gain mechanistic insight into age-dependent, WOS-specific chromatin looping on a genome-wide scale, we generated Hi-C (Lieberman-Aiden et al., 2009) and RNA-seq data for rats within WOS and outside WOS. We compared the temporal changes in looping to those in expression and found that genes up-regulated within the WOS are associated with interactions that are higher within WOS, and a similar trend exists for genes outside the WOS. We developed a computational approach that combined network enhancement and non-negative matrix factorization (NMF) to identify “dynamic” blocks representing larger scale topological changes between the two time points. We found that network enhancement was important for reliable detection of dynamic blocks, many of which harbored genes implicated in cancer-related pathways and processes. Finally, we mapped human breast cancer GWAS SNPs to loci in rat and found conserved interactions with genes between human and rat. Taken together, these results identified individual loop level and larger-scale topological differences between within-WOS and outside-WOS, many of which are related to transcriptional differences.

## Methods

### Tissue Collection and Hi-C Assay

Fresh mammary glands from the abdominal/inguinal regions of 6-week-old and 12-week-old female mammary cancer susceptible Wistar-Furth rats were individually collected, scissor minced and digested for 2 h at 37°C in 10 ml of GIBCO Dulbecco’s modified Eagle’s medium/F12 (DMEM/F12; ThermoFisher) containing 0.005 g/ml of type 3 collagenase (Worthington-Biochem). Centrifugation was used to remove fat and collect the cell pellets. Individual cell pellets were washed and resuspended in DMEM/F12 media. Each cell suspension was filtered using 40 μm nylon to enrich the mammary ductal fragments and remove stromal cells. The filter was inverted and rinsed to collect the fragments, and the resulting cell pellet containing mammary epithelial cells (MECs) was diluted in PBS and treated for 10 min with 1.5% formaldehyde for DNA/chromatin fixation. After a series of washes, the final cell pellets were collected using centrifugation and stored at −80°C. A total of 6 samples were sent to Arima Genomic, Inc. (*n* = 3 for 6-week-old and *n* = 3 for 12-week-old) for Hi-C analysis, consisting of complete sample processing for Hi-C and library preparation and Illumina Next-Generation sequencing.

### Hi-C Data Processing and Differential Interactions

Hi-C data was generated with ∼430 M reads per replicate. Hi-C reads were processed using HiC-Pro version 2.7 (Servant et al., 2015) with the default BowTie2 parameters (--very-sensitive -L 30 --score-min L,-0.6,-0.2 --end-to-end–reorder) and aligned to the rn6 genome and 10 kb contact maps were generated. The 6 and 12 weeks samples were aggregated to one 6 weeks and one 12 weeks contact count matrix, respectively. HiC-Pro’s implementation of ICE normalization (Imakaev et al., 2012) with default parameters was performed on the two resulting Hi-C matrices.

In order to determine a set of differential chromatin interactions (DCIs), we used both Selfish (Ardakany et al., 2019) and Fit-Hi-C (Ay et al., 2014). Selfish uncovers DCIs between two contact maps directly using a novel self-similarity measure (Ardakany et al., 2019). We obtained 453,513 differential interactions from Selfish (p-value cutoff 10^−4^). We also used Fit-Hi-C with one pass and a mappability threshold of 1 to determine significant interactions (q-value < 0.05) within WOS and outside WOS. We took differential interactions as those that were significant in one but not the other, resulting in 1,306,601 interactions. We took the union of the resulting FitHiC and Selfish interactions to generate a total of 1,447,082 interactions. We filtered these interactions by computing the mean and standard deviation for all within WOS DCI and all outside WOS DCIs separately for each distance bin (bins at 50 kb intervals from 0 to 1 Mb). Only differential interactions with a z-score greater than one and a distance equal to or less than 1 Mb were considered, for a final set of 1,072,652 interactions. The Fit-Hi-C approach tended to yield pairs with greater differences at longer distances while Selfish tended to yield pairs with greater differences at shorter differences (Supplementary Figure S1 **)**.

### Tissue Collection, RNA Extraction and RNA-seq Experiments

To examine the transcriptional differences associated with WOS on a genome-wide scale, we measured gene expression levels in the MEC from 6-week-old (entering WOS) and 10-week-old (exiting WOS) susceptible rats (*n* = 6 for 6-week-old, *n* = 7 for 10-week-old) using RNA-seq following a similar protocol as described in Henning et al. (2016). Briefly, mammary glands were removed, minced, digested with collagenase, followed by differential centrifugation to collect mammary ductal organoids, which are mainly composed of epithelial cells along with stromal fibroblasts and immune cells. To isolate RNA, cells were homogenized in TRI Reagent (Ambion), followed by RNA extraction using the MagMAX-96 for Microarrays Total RNA kit (Ambion). RNA was extracted using the RNeasy Mini Kit (Qiagen). The fastq files were processed by the UW Biotech center. Counts were obtained using RSEM v1.2.22 (Li and Dewey, 2011).

For all samples, we calculated Transcripts per Million (TPM) for 14,792 genes in the rat genome using RSEM v1.2.22 (Li and Dewey, 2011). We applied several algorithms to determine differential expression: DESeq (Love et al., 2014), EBSeq (Leng et al., 2013) and EdgeR (Robinson et al., 2010). EBSeq was the most conservative with 461 genes. DESeq (3401) and EdgeR (2547) had an intersection of 2071 genes (Supplementary Figure S2). We therefore took EBSeq plus the intersection of DESeq and EdgeR as the total set of 2533 differentially expressed (DE) genes.

### Network Enhancement and Multiview Non-Negative Matrix Factorization

We developed the NE-MVNMF approach to analyze multiple Hi-C datasets. NE-MVNMF applies Network Enhancement (NE) followed by Multiview Non-negative Matrix Factorization (MVNMF) (Liu et al., 2013) to our Hi-C datasets.

Network Enhancement (NE) is a method for denoising a biological network (Wang et al., 2018). We consider a Hi-C dataset as a weighted network of genomic regions, where each node in the network corresponds to each genomic region and the weighted edges connecting the nodes represent the interaction frequency between genomic regions. NE takes a noisy Hi-C matrix as input and applies iterative graph diffusion process to strengthen edge weights that are well-supported by strong neighboring edges and weaken poorly supported edges. The output of NE is a denoised, enhanced, symmetric matrix which can be used as input to the next step in our pipeline, MVNMF.

Multiview Non-negative Matrix Factorization (MVNMF) is a multi-task non-negative factorization (NMF) method which allows us to find a common underlying structure in multiple matrices (Liu et al., 2013), each task corresponding to a matrix. MVNMF does this by finding low-dimensional factors of multiple matrices such that the factors are regularized towards a common consensus. These factors can then be used as latent features for clustering to reveal the underlying shared or divergent structure in the data. Formally, given t ∈{1,…,T} tasks, each with input matrix $X^{\left(t\right)}\in {\mathbb{R}}_{\geq 0}^{n_{t}\times m}$ , the objective is to find task-specific factors $U^{\left(t\right)}\in {\mathbb{R}}_{\geq 0}^{n_{t}\times k}$ , $V^{\left(t\right)}\in {\mathbb{R}}_{\geq 0}^{m\times k}$ and the consensus factor $V^{\left(c\right)}\in {\mathbb{R}}_{\geq 0}^{m\times k}$ such that:

$$min_{U^{\left(t\right)},V^{\left(t\right)},V^{\left(c\right)}}\sum_{t=1}^{T}{\left\|X^{\left(t\right)}-U^{\left(t\right)}V^{\left(t\right)T}\right\|}_{F}^{2}+\alpha {\left\|V^{\left(t\right)}-V^{\left(c\right)}\right\|}_{F}^{2}$$

Here k , is the number of factors or reduced dimensions and is much smaller than $n_{t}$ or m. The regularization term, α , will constrain factor $V^{\left(t\right)}$ of task  t to be similar to the consensus $V^{\left(c\right)}$ . Liu et al. originally proposed an iterative multiplicative update algorithm for MVNMF. However, multiplicative updates algorithm is often slow to converge. Therefore, we implemented an algorithm that optimizes this objective using hierarchical alternating least squares (HALS) with convergence guarantee to a local minimum (Kim et al., 2014).

In our application of MVNMF, we have two tasks, each corresponding to an input Hi-C matrix at 10 kb resolution, for each chromosome: one matrix from week 6 and another one from week 12. The rows and columns of this matrix correspond to a 10 kb bin. Since intra-chromosomal Hi-C matrices (as well as their network-enhanced versions) are symmetric, $X^{\left(t\right)},\;U^{\left(t\right)},\;V^{\left(t\right)},\;V^{\left(c\right)}$ take on the dimensions of n×n, n×k, n×k , and n×k , respectively.

We use a simple heuristic to pick k , the number of the factors, which also is the number of clusters. Based on our previous work on single-task NMF to Hi-C data, we set k such that the expected size of each cluster is about 1 Mb in length, which corresponds to the average size of TADs (Lee and Roy, 2021). For example, for an input matrix that corresponds to a chromosome of size 10 Mb, we set k=10. Here, we used 56-282 factors to capture TAD like structures, corresponding to the size of the rat chromosomes.

We verified that network enhancement (NE) and downstream NMF does not overcorrect the underlying structure of the input matrix by comparing NMF results on Hi-C data of different depths. Briefly, we first downsampled high-depth Hi-C matrices from the GM12878 cell line (Rao et al., 2014) to four different lower depth levels (equivalent to the read depth of cell lines HMEC, HUVEC, NEHK, and K562 from the same study). We then applied NE to the downsampled matrices, and then applied NMF to the original high-depth matrices, downsampled matrices, and downsampled + NE matrices. When we compared to original Hi-C data, downsampled Hi-C data + NE does not lead to significant differences in the number of regions in each cluster Supplementary Figure S3A). However, downsampled + NE does lead to significantly larger number of regions in each cluster when compared to downsampled without NE (t-test p-value < 0.05 for all downsampled depths). Additionally, when compared to the original data, downsampled + NE does not lead to significant differences in the length of contiguous regions with the same cluster assignment (Supplementary Figure S3B). Furthermore, we measured the similarity of the clustering results from the high-depth matrices to those from the downsampled matrices, as well as between the high-depth matrices and the downsampled + NE matrices. The cluster similarity was measured with Rand Index, which measures the concordance between a pair of clustering results. Rand Index ranges from 0 to 1; Rand Index value of 1 means all data points found in one cluster in one result are also in one cluster in the other result, and those in distinct clusters in one result are also kept separate in the other result. We find that the cluster similarity between the original high-depth matrices versus the downsampled matrices is comparable to the cluster similarity between the original and NE matrices (Supplementary Figure S3C), suggesting that NE does not overcorrect the underlying structure of the data.

MVNMF, like NMF can converge to different local optima. Therefore, we verified the stability of our results to different random initializations. Briefly, we applied MVNMF to chromosomes 7, 11, 15 and 19 on the within-WOS and outside-WOS matrices with 5 different random initialization seeds. We evaluated the stability of the clusters from different random initializations using Rand Index. We measured the Rand Index between every pair of clustering results from different random initialization seeds (Supplementary Figure S4). We find that the mean Rand Index across these comparisons is around 0.9 suggesting that the clustering results are stable to the random initialization seeds.

### Identification of Static *Versus* Dynamic Blocks

Once we have the factors, we use them to identify genomic regions dynamically changing their interaction profile across tasks, which we refer to as “dynamic blocks.” First, we assign all regions to a cluster based on the factors from MVNMF, then find regions whose cluster assignment changes. We take advantage of the fact that column j in $V^{\left(t\right)}$ of task t corresponds to the latent feature or column j in $V^{\left(s\right)}$ of task s . Since $X^{\left(t\right)}$ is symmetric in Hi-C data, either $U^{\left(t\right)}$ or $V^{\left(t\right)}$ can be used to define the clusters of regions. Assuming we use $U^{\left(t\right)}$ , we assign each row i (corresponding to genomic region i ) to its most dominant latent feature, $c_{i}^{\left(t\right)}=argmax_{j\;\in \left\{1,\ldots ,k\right\}}U^{\left(t\right)}\left[i,j\right]$ , where $U^{\left(t\right)}\left[i,j\right]$ represents the entry in the i th row and the j th column/latent feature of $U^{\left(t\right)}$ . We repeat this procedure across all tasks. A dynamic block between task t and s is a contiguous stretch of 10 kb regions, at least 50 kb in length, whose cluster assignment changed between them, i.e., $c_{i}^{\left(t\right)}\neq c_{i}^{\left(s\right)}$ . Furthermore, all regions within the block have to have the same cluster ID within a task. Conversely, a static block is one where $c_{i}^{\left(t\right)}=c_{i}^{\left(s\right)}$ . To further assess if a dynamic block is indicative of a changing interaction frequencies, we compared the count differences within the block across time points. We expect a dynamic block to exhibit significantly greater count differences compared to static blocks. We further verified these trends using a t-test to assess the difference in counts between time points among regions inside a static block as well as among regions inside a dynamic block.

### Human GWAS Study Integration

We downloaded supplementary table S15 from Zhang et al. which contains gene-SNP interactions from the INQUISIT software (Zhang et al., 2020) and supplementary table S5 from Baxter et al. which contains Capture Hi-C SNP-gene associations (Baxter et al., 2018). We used liftOver (Kuhn et al., 2013) to map these SNPs from the human hg19 assembly to a locus in the rn6 rat assembly, with a minimum base overlap ratio of 0.1. Since the position of the SNP in human may not be a SNP in the corresponding lifted over position in rat, we refer to the position as a ‘locus’ in rat. We intersected the rat locus with differential chromatin interactions (DCIs) by checking if the SNP was within the boundary of either 10 kb bin in the interaction. We mapped the other 10 kb end to a gene if it overlapped any 10 kb bin within the genomic coordinates of the gene provided by Ensembl release 96 (Yates et al., 2020). We referred to resulting gene locus pairs as locus-gene DCIs. We used the common names to match genes from human to rat.

## Results

### Differential Looping is Associated With Differential Expression of Within WOS Versus Outside WOS Rats

**FIGURE 1:**
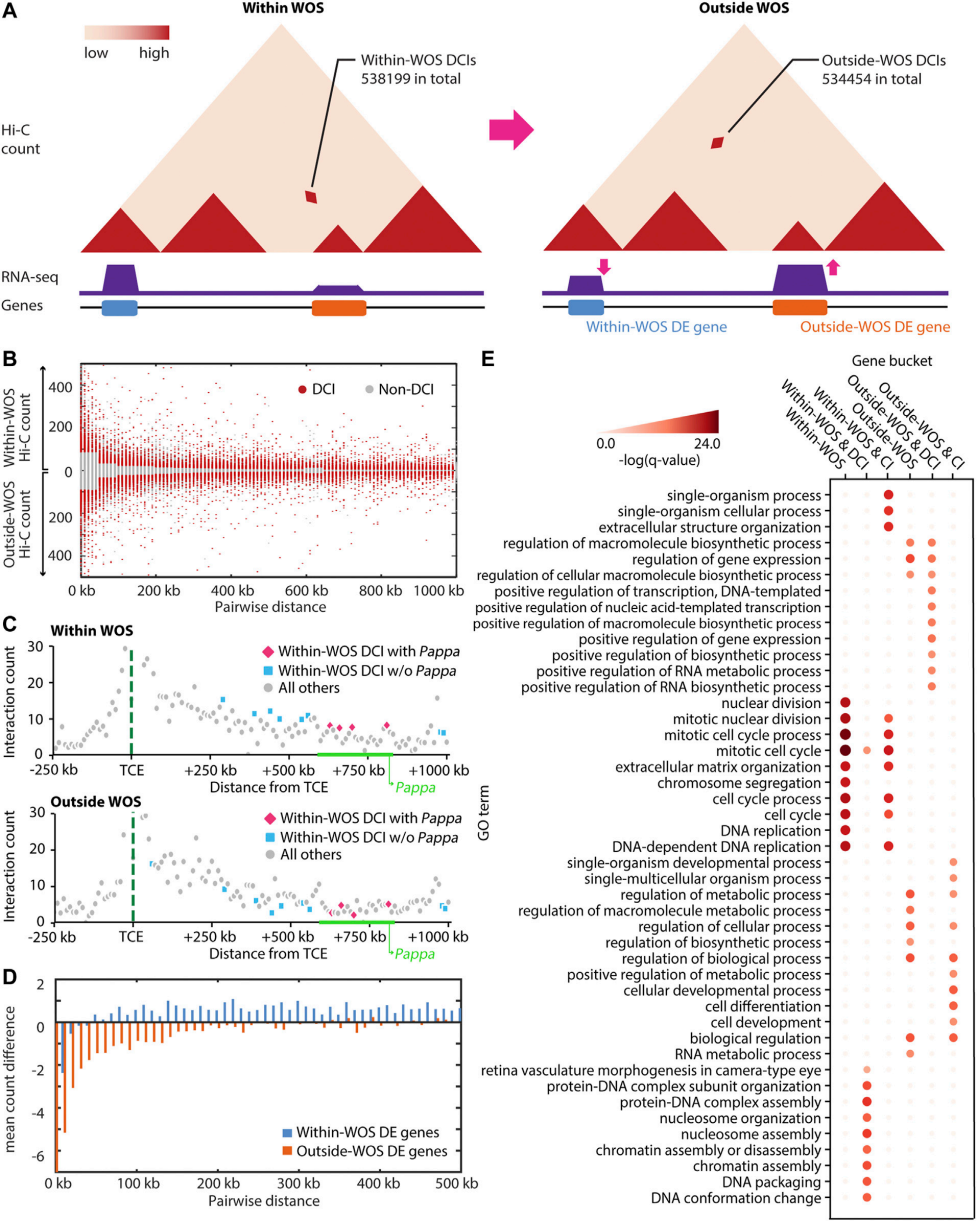
Characterizing WOS-specific chromatin interactions and gene expression. **(A)** Hi-C and RNA-seq data was generated to globally characterize the three-dimensional genome organization and transcriptome within and outside the window of susceptibility (WOS). We first characterized these changes at the level of individual interactions or loops. Within WOS, we identified 538,199 differential chromatin interactions (DCIs) across all chromosomes and 534,454 DCIs outside the WOS. Here CIs refer to those pairs with significantly high counts within and/or outside WOS, whereas DCIs refer to interactions exclusively higher in one of within-WOS or outside-WOS context. **(B)** Contact counts for DCIs (red) compared to non-DCI (gray) for within WOS (above *x*-axis) and outside WOS (below *x*-axis) in rat chromosome 1. **(C)** Visualization of contact counts a −250 kb to +1 Mb around the temporal control element (TCE, green dotted line) within WOS **(top)** and outside WOS **(bottom)**. We plot the interaction count between the TCE region and neighboring regions by distance. Blue and pink dots are DCIs that are higher within WOS. Gray dots are all others. Pink dots are additionally associated with the *Pappa* gene. **(D)** Mean count difference (within-WOS count – outside-WOS count) for Fit-Hi-C significant interactions associated with genes that are up-regulated within WOS (i.e., within-WOS DE genes, blue) and up-regulated outside WOS (outside-WOS DE genes, orange). **(E)** Gene Ontology (GO) enrichment of DE genes within/outside WOS, those associated with DCIs, and those associated with significant chromatin interactions (CI). Intensity of red is associated with the -log (q-value) of the GO enrichment.

To globally characterize chromatin looping and examine its role in establishing WOS and associated gene expression programs, we generated Hi-C and RNA-seq datasets for rat mammary epithelial cells within WOS (6-weeks) and outside WOS (10-weeks RNA-seq, and 12-weeks Hi-C, Figure 1A). Hi-C data was generated with ∼430 M reads per replicate. We aggregated reads to 10 kb resolution and used Iterative Correction and Eigen vector decomposition (ICE) (Servant et al., 2015) to normalize the Hi-C matrices from the two time points (Methods). We used ICE for normalization because it is recommended for Fit-Hi-C (Ay et al., 2014), however, our approach is applicable to data from other normalization methods as well (Hu et al., 2012).

We first identified differential chromatin interactions (DCIs) between the WOS and outside the WOS by taking the union of results from two approaches: Selfish (Ardakany et al., 2019) and the difference in significant chromatin interactions (CIs) identified by applying Fit-Hi-C individually to each sample (Ay et al., 2014). We then filtered these DCIs based on a distance-stratified absolute value of z-score of 1.0 (Methods) to focus on the differential interactions with the greatest magnitude of change in and out of the WOS (Figure 1B). In total we identified 538,199 DCIs with counts higher in the WOS (within WOS DCIs) and 534,454 DCIs with counts lower in the WOS compared to outside the WOS (outside WOS DCIs). Among the DCIs, we recapitulated several TCE-*Pappa* gene interactions that are higher in the WOS compared to outside the WOS (Figure 1C), which is consistent with previous observations that the TCE interacts with *Pappa* in a WOS-dependent manner (Henning et al., 2016). In parallel, we applied DESeq2 (Love et al., 2014), EBSeq (Leng et al., 2013) and EdgeR (Robinson et al., 2010) to identify differentially expressed (DE) genes between the WOS and outside the WOS (FDR corrected p-val < 0.05, Methods). In total we identified 2300 DE genes, 1,358 of which were up-regulated within the WOS and 942 were down-regulated within the WOS compared to outside.

To examine the relationship between differential expression and chromatin organization we linked DE genes, regardless of direction of expression change, to Fit-Hi-C CIs either in and/or out of the WOS (Figure 1D; Table 1). We computed the average difference in contact count, stratified by distance, for genes upregulated in WOS compared to outside. We found that at all distance bins compared, genes upregulated in WOS have a higher average contact count in WOS compared to outside WOS for significant interactions (t-test p-value = 3.8e-107, Figure 1D). Likewise, genes upregulated outside WOS (or downregulated in the WOS) have a higher average contact count outside WOS compared to within WOS. We performed a similar analysis for DCIs that are significant within or outside WOS and found a similar result (Supplementary Figure S5). These results show that changes in gene expression between the two time points is in part due to differences in 3D genome organization.

**TABLE 1:** Number of interactions associated with genes differentially up-regulated within WOS and outside WOS.

	# Of within WOS genes (total = 1,358)	# Of interactions assoc. With within WOS genes	# Of outside WOS genes (total = 942)	# Of interactions assoc. With outside WOS genes
FitHiC interactions	1,331	118,169	925	151,845
DCI interactions (total)	1,310	45,385	919	59,158
DCI interactions (within WOS)	1,245	23,591	886	27,257
DCI interactions (outside WOS)	1,262	21,794	907	31,901

We next examined the biological processes associated with WOS-specific changes. Genes that are up-regulated within the WOS (1,358 genes) are enriched for cell cycle and DNA replication (Hypergeometric test with FDR<0.05, Figure 1E). We also examined genes that are up-regulated in the WOS and associated with 5 or more within WOS DCIs (355 genes). We chose this threshold since many DE genes are associated with at least one DCI in WOS (Table 1). Genes that are up-regulated within the WOS and are additionally associated with 5 or more DCIs in the WOS are enriched for these processes as well as DNA packaging and conformation. Genes that are up-regulated outside the WOS (942 genes) are enriched for general transcriptional regulation, and RNA metabolism processes. Genes up-regulated outside WOS and interacting with 5 or more outside WOS DCIs (316 genes) were enriched for similar terms. We also found that within WOS upregulated genes with long-range interactions were enriched for similar terms as all upregulated genes within the WOS, while genes upregulated within WOS and interacting with DCIs were enriched with markedly different terms compared to all upregulated genes in the WOS (Figure 1E). Taken together, these results suggest that DCIs are likely involved with the regulation of cell cycle and DNA packaging in younger rats, while in mature rats, DCIs may be more involved with maintaining transcriptional control.

### Matrix Factorization-Based Approach to Examine Higher Order Organization Dynamics

**FIGURE 2:**
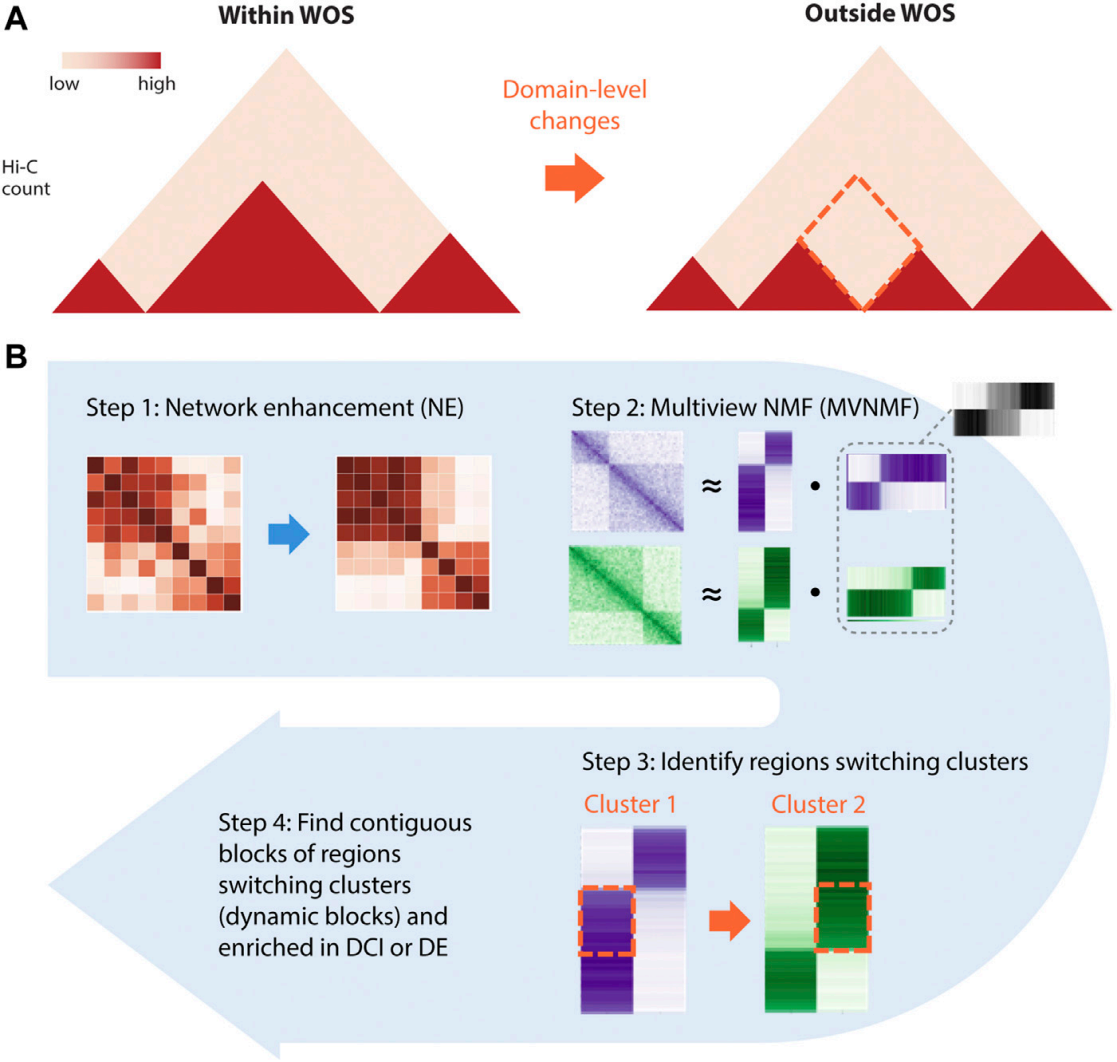
Overview of NE-MVNMF. **(A)** The goal of NE-MVNMF is to find higher-order, e.g., TAD-level changes between the two timepoints denoted as red-dashed diamond. **(B)** Steps of NE-MVNMF. First, network enhancement (NE) smooths out the within-WOS and outside-WOS Hi-C matrices. Then MVNMF is applied to jointly factor the two matrices. By clustering the factor matrices, regions that are switching cluster assignments between within and outside WOS can be identified. These contiguous blocks of such regions represent domain-level changes which we call “dynamic blocks.” On the dynamic blocks, we do downstream analysis, such as check for association with DCIs or DE genes.

In addition to changes at the level of individual interactions, higher-order structural changes in chromatin organization within and outside the WOS could be important for molecular changes associated with breast cancer susceptibility. However, identification of higher-order structural changes, such as in TADs, poses two challenges: (1) handling the noise and sparsity in the input Hi-C matrices and (2) the difficulty in matching TADs across datasets or conditions so that changes between them can be pinpointed. To address these two challenges, we developed and applied an approach, Network Enhancement with Multi-view Non-negative Matrix Factorization (NE-MVNMF), which first applies network enhancement (NE) to smooth the noisy and sparse Hi-C matrices (Wang et al., 2018) and then performs multi-view non-negative matrix factorization (MVNMF) (Liu et al., 2013) on these matrices to identify large-scale conserved and differential structural units (Figure 2, Methods).

The first component of our pipeline, network enhancement (NE) was developed originally for denoising biological networks (Wang et al., 2018) (Figure 2B, Step 1). In our application, we treat a Hi-C dataset as a weighted network of chromatin regions: each node in the network corresponds to a region and the edge weights between nodes represent the interaction counts between a pair of regions. NE iteratively enhances edge weights that are well-supported by strong neighboring edges and weakens those that are poorly supported, then outputs a denoised matrix which is then used as input to the next step in our pipeline, MVNMF. We verified that NE does not overcorrect the underlying structure of the input matrix by comparing results on Hi-C data of different depths before and after smoothing (Supplementary Figure S3, Methods).

**FIGURE 3:**
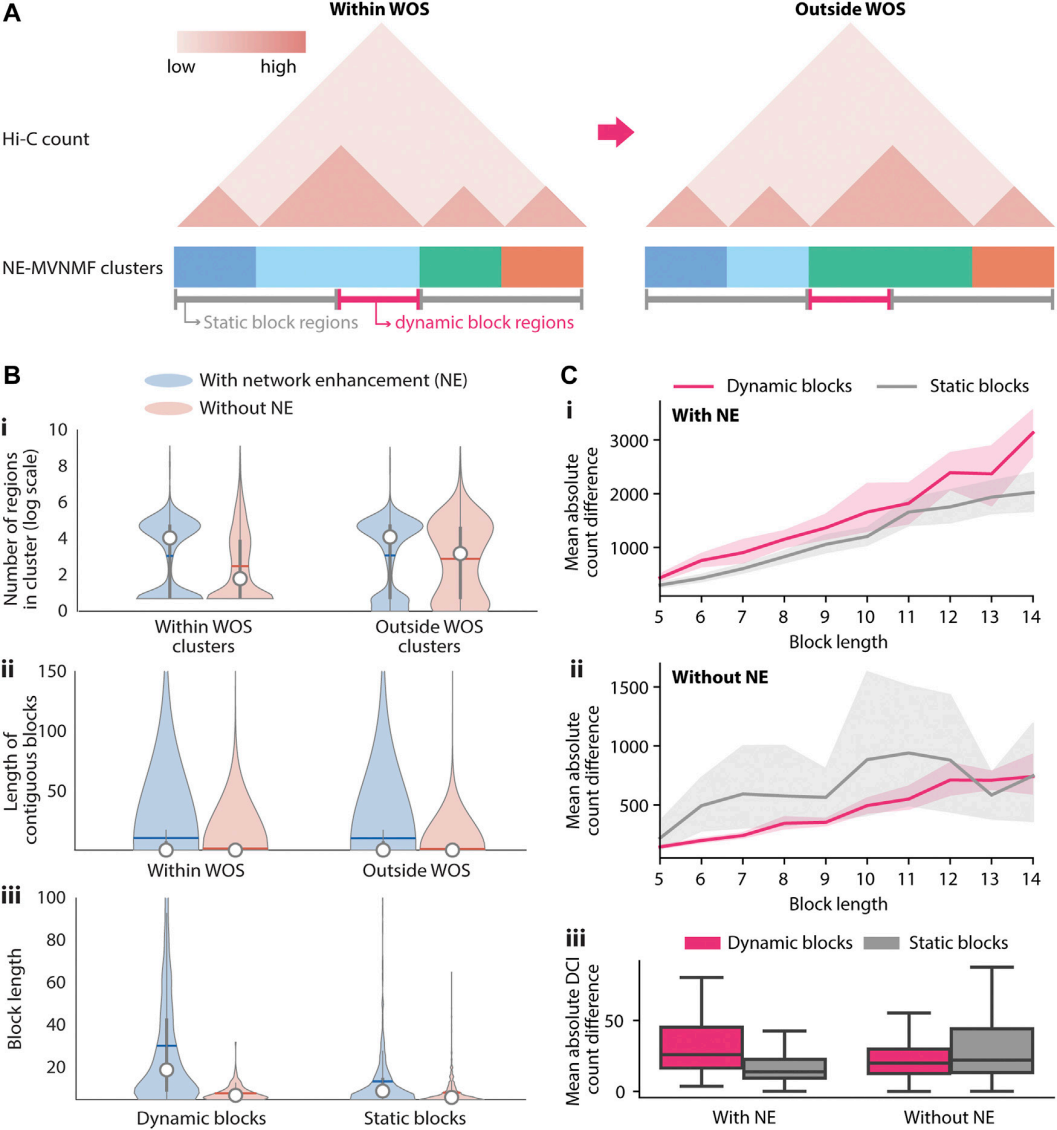
Identification and characterization of dynamic 3D genome blocks with NE-MVNMF. **(A)** Schematic of how dynamic blocks of regions involved in large-scale topological changes are identified from the NE-MVNMF clusters. The NE-MVNMF clusters are depicted at the bottom of the exemplar Hi-C count matrix, with regions in the same cluster to have the same color. Regions in dynamic blocks (magenta line) are regions whose cluster assignment switched between within WOS and outside WOS. Conversely regions in static blocks (gray) are those whose cluster assignment stayed the same. **(B)** Distribution of the number of regions in each cluster within and outside WOS (i), length of contiguous blocks within and outside WOS (ii), and length of contiguous dynamic or static blocks (iii), with and without NE. **(C)** Difference in interaction counts among regions within dynamic blocks and static blocks. Top, for each dynamic or static block, we summed up the absolute value difference between interactions from within WOS and those form outside WOS. We plot the mean of the absolute value difference by block length with NE (i) and without NE (ii). The shaded area represents the 95% confidence interval. iii, Mean absolute value difference for DCIs only is plotted for dynamic and static blocks, with and without NE (iii).

MVNMF combines Non-negative Matrix Factorization (NMF) (Lee and Seung, 2001) with multi-task learning (Zhang and Yang, 2021). NMF is a powerful dimensionality reduction method that can be used to recover interpretable, lower-dimensional patterns from large, high-dimensional data in imaging, text, and biomedical domains (Lee and Seung, 2001). Applying NMF to a Hi-C matrix yields low-dimensional factors or latent features which can be used to cluster the row or the column entities, i.e., genomic regions. These clusters of genomic regions correspond to densely interacting regions of the genome such as topologically associating domains (TADs) (Lee and Roy, 2021). MVNMF extends NMF to a multi-task setting with multiple input matrices, each corresponding to a task or a time point (e.g. within WOS). It jointly factorizes the input matrices such that their lower-dimensional factor representations are similar to a single common factor, and clusters derived from these factors can be matched across tasks (Methods). MVNMF identifies clusters that are matched across tasks. This cluster correspondence across tasks allows us to easily identify genomic regions whose cluster assignment has changed in different contexts (Figure 2B, Step 2). From these matched clusters, we define a “dynamic block” as a stretch of 5 or more contiguous 10 kb bins (50 kb region) that have a different cluster assignment between a pair of conditions (Figure 3A). A “static block” is similarly defined but for contiguous 10 kb bins that have the same cluster assignment between the conditions compared. Regions that do not have contiguous cluster assignments for 5 or more regions are considered noisy.

We first compared the effect of network enhancement on the ability to detect higher-order topological units and the quality of the dynamic versus static blocks based on different metrics. We applied both MVNMF and NE-MVNMF at different regularization values ( α ϵ {1e5, 1e6, 1e7, 1e8} Methods) to within and outside the WOS Hi-C matrices at 10 kb resolution. Higher regularization values will constrain the factors from each task to be more similar to the consensus factor (Methods). We examined the distribution of the cluster sizes with and without NE (Figure 3Bi), the extent to which they were contiguous (Figure 3Bii) and the distribution of sizes of static and dynamic blocks (Figure 3Biii). When comparing the overall size of the clusters, without NE tends to obtain on average “smaller” units compared to with NE (Figure 3Bi). Furthermore, when we consider the number of contiguous regions with the same cluster assignment before reaching a different cluster assignment, the lengths of such contiguous regions are larger for NE versus no NE in both within and outside WOS (Figure 3Bii). When comparing the dynamic versus static blocks, we see a similar trend in the size distribution (Figure 3Biii): network enhancement and higher levels of regularization tended to increase the number of static blocks and decrease the number of dynamic blocks overall (Supplementary Table S1). These results show that network enhancement led to more contiguous larger blocks, which are indicative of less noisy clustering assignments. Finally, we hypothesized that there would be a larger difference in overall contact count in dynamic blocks compared to static blocks. We took the overall difference for each block as the sum of the absolute value of the difference for all contact counts within WOS and outside WOS. We compared these count differences for blocks of different sizes (Figures 3Ci,ii) as well as across all blocks (Figure 3Ciii). We find that for the same α regularization value, dynamic blocks have a greater overall difference than static blocks when using network enhancement (Figure 3C for α=1e8 , Supplementary Figure S6 for α ϵ {1e5, 1e6, 1e7} ). For blocks with 3 or more regions, the overall difference for dynamic blocks was significantly greater than static blocks for 6 out of the 10 bins (t-test p-value <0.05). When comparing across blocks of all sizes, the count difference of DCIs between static and dynamic blocks was much more dramatic compared to without NE (Figure 3Ciii). Taken together, NE-MVMF allows us to reliably identify regions involved in higher-order topological changes across multiple biological contexts.

### NE-MVNMF Reveals Large-Scale WOS-Specific Changes

**FIGURE 4:**
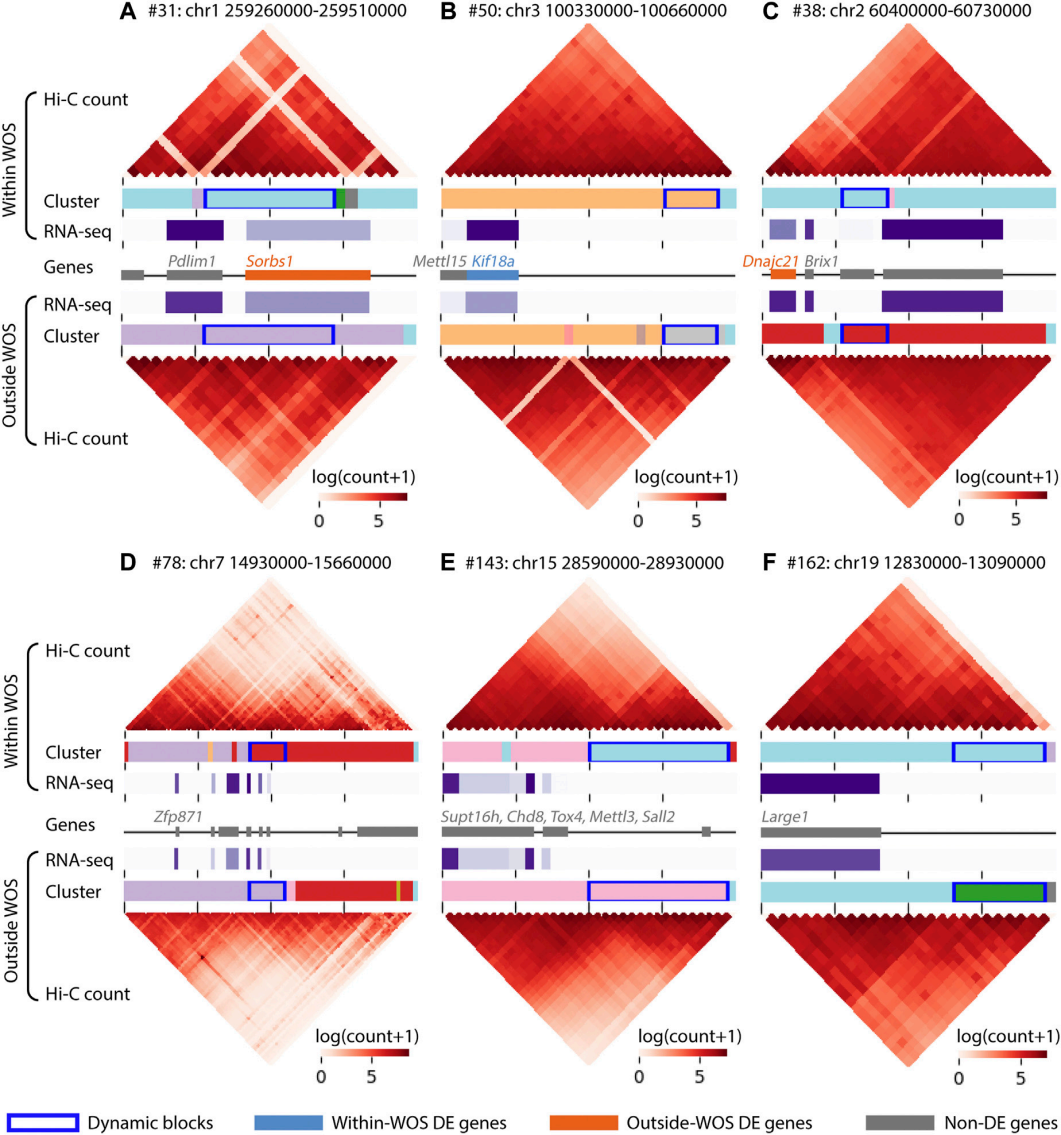
Visualization of regions surrounding 6 dynamic blocks of interest in the window of susceptibility. Within each panel, the top and the bottom heatmaps visualize the interaction counts from within and outside WOS, respectively. The horizontal bars associated with Cluster below each heatmap are colored by cluster ID; dynamic blocks are highlighted with a dark blue box outline. Gene expression (RNA-seq in TPM) is visualized in a horizontal purple heatmap, with darker purple representing higher expression. Finally, the gene track in the middle denotes gene locations; within-WOS and outside-WOS DE genes are colored with blue and orange, respectively. Gray indicates the gene is not DE. The dynamic blocks of interest are found within **(A)** #31, chr1 259260000-259510000, **(B)** #50, chr3 100330000-100660000, **(C)** #38, chr2 60400000-60730000, **(D)** #78, chr7 14930000-15660000, **(E)** #143, chr15 28590000-28930000, **(F).** #162, chr19 12830000-13090000.

We next examined the dynamic blocks obtained from NE-MVNMF to gain insight into the 3D genome organizational properties within and outside the WOS. There were 168 dynamic blocks in total across all chromosomes, which ranged in size from 50 to 320 kb. We prioritized blocks for interpretation based on the difference in counts of blocks between the two conditions as well as based on visual inspection**.** Of the total 168 blocks, we identified 35 blocks that had a significant change in count between the two conditions when considering all pairs of regions in these blocks (T-test and Rank sum *p*-value <0.05). These blocks ranged from 50 to 180 kb in size and included blocks spanning genic regions (28) and those spanning non-coding regions (7, Figure 4 and Table 2).

**TABLE 2:** Number of genic and non-genic dynamic blocks. A genic block is one which has genes.

	All blocks	Significant (t-test and rank sum)
Total	168	35
Genic	95	28
Non-genic	73	7

For ease of interpretation, we focused on blocks that harbored genes, regardless of their differential expression status. For example, one block (#31,110 kb, Figure 4A), included the genes *Pdlim1* and *Sorbs1*, of which *Sorbs1* exhibited a significantly lower expression within WOS (6 weeks), while *Pdlim1* exhibited a relatively higher, expression in the WOS. *Pdlim1* is expressed in fibroblasts and involved in cell polarity and migration (Stelzer et al., 2016) and has been shown to be associated with breast cancer progression (Liu et al., 2015). *Sorbs1* is involved in signaling pathways and low expression of *Sorbs1* is associated with poor prognosis of breast cancer (Song et al., 2017). Another block (#50, 60 kb, Figure 4B) spanned two genes, *Kif18a* and *Mettl15* of which *Kif18a* has a significantly high expression within WOS. *Kif18a* is a kinesin protein involved in chromosomal stability, low expression of kinesin proteins has been associated with cell proliferation of chromosomally unstable genes (Marquis et al., 2021) and is a candidate target for cancer treatment (Sabnis, 2020). Another block harboring a down-regulated gene within the WOS was #38 (50 kb, Figure 4C), containing *Dnajc21* a heat shock protein and *Brix1* involved in ribosome biogenesis. Over-expression of heat shock proteins has been associated with a large number of cancers (Calderwood and Gong, 2016). Another block (#78, 90 kb, Figure 4D) was associated a number of zinc finger proteins, including *Zfp871*, which was shown to be part of the P53 pathway (Mohibi et al., 2021) and cytochrome P450, an enzyme that metabolizes several pre-carcinogens and is broadly involved in both cancer formation and treatment (Rodriguez-Antona and Ingelman-Sundberg, 2006). Finally, blocks 143 (160 kb, Figure 4E) and 162 (80 kb, Figure 4F) had several genes that either encoded chromatin remodeling factors (*Supt16h* and *Chd8*), genes representing families often mutated in cancers (*Tox4*, Yu and Li, 2015), genes that have been implicated as oncogenes as well as tumor suppressors (*Sall2*, Hermosilla et al., 2017; *Mettl3*, Zeng et al., 2020) and involved in glycosylation (*Large*1, block162) which is used as a marker and offers novel therapeutic targets (Costa et al., 2020). Overall, our analysis identified dynamic blocks that harbored genes implicated in cancer and related pathways including chromosomal stability, ribosome biogenesis and stress response.

### WOS-Specific Looping can be Leveraged to Examine Regulatory Variation

**FIGURE 5:**
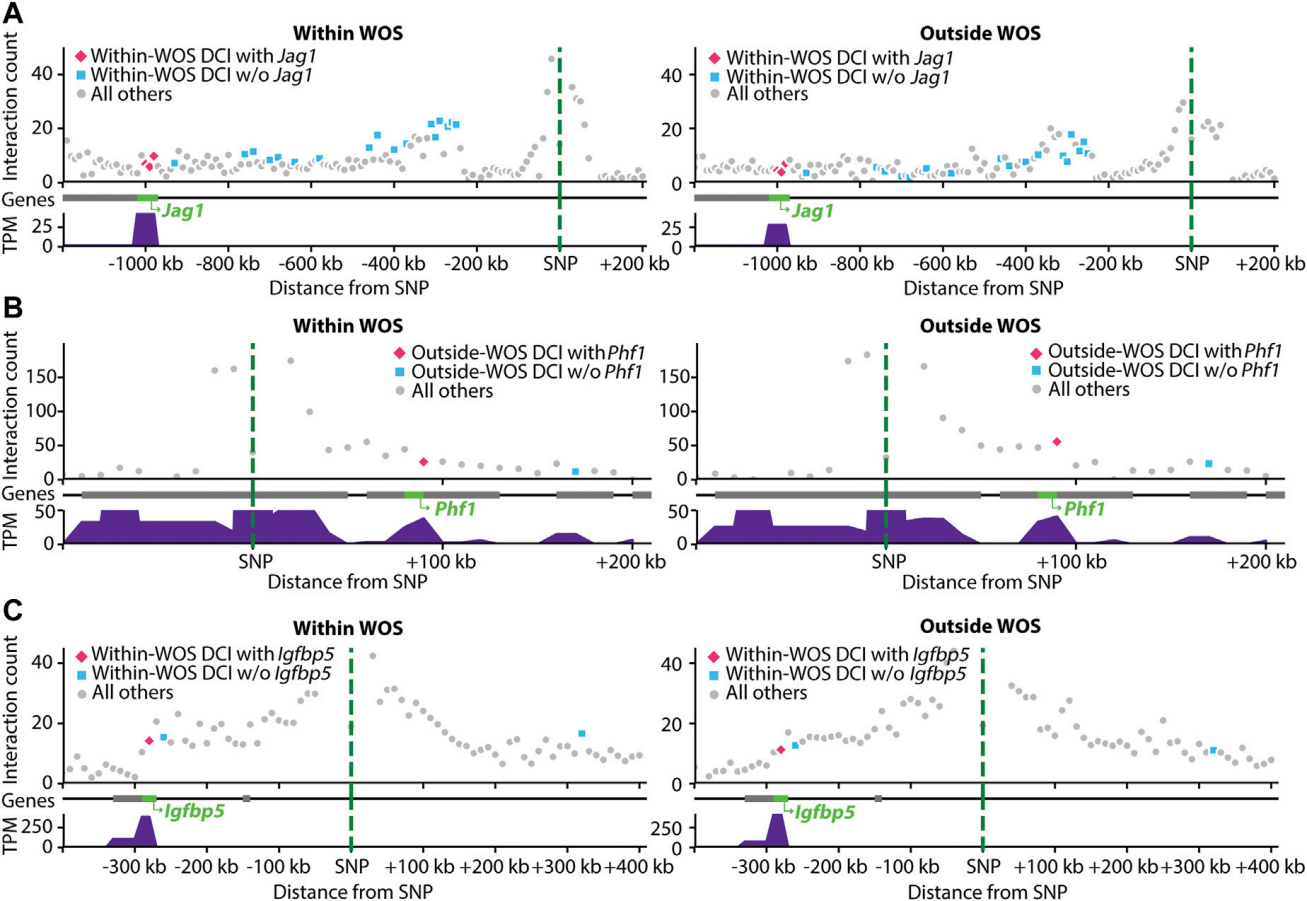
Visualization of interactions involving SNPs associated with human breast cancer mapped to rat genome. Within each panel, shown are the interaction counts from within WOS **(left)** and outside WOS **(right)** between the mapped-SNP region (green dotted line) and neighboring regions by distance. Pink dots are DCIs associated with the gene of interest; blue dots are other DCIs; gray dots are all others. Gene tracks denote gene locations; the gene of interest is highlighted in green. Gene expression (in TPM) is plotted below the gene tracks. **(A)** Interactions involving human breast cancer SNP (chr20 11502618 A- > AAC) mapped to a region in rat. The region is involved in within-WOS DCI with the gene *Jag1*. **(B)** Interactions involving human breast cancer SNP (chr6 33239869 C- > T) mapped to a region in rat. This region is involved in outside-WOS DCI with gene *Phf1*. **(C)** Interactions involving human breast cancer SNP (chr3 156535958 AT- > A) mapped to a region in rat. This region is involved in within-WOS DCI with gene *Igfbp5*.

Many studies have identified disease associated variants inside distal regulatory elements that loop to genes, for example, in autoimmune disorders (Javierre et al., 2016) and cancer (Zhang et al., 2019) including breast cancer susceptibility (Baxter et al., 2018). What is less explored is the context-specificity and timing of these long-range interactions, which can impact when a variant modulates a target gene’s expression. The Temporal Control Element (TCE) interaction with the *Pappa* gene is an example of a time window-specific interaction and is present in young rats (within WOS), but not older rats (Henning et al., 2016). In the susceptible genotype, *Pappa* expression levels are increased relative to the resistant genotype leading to increased breast cancer susceptibility, indicating a genotype-specific effect on gene expression. A similar model could underlie other SNPs associated with breast cancer susceptibility, where the SNP occurs in an enhancer region that loops to regulate a gene’s expression in a condition-specific manner (e.g. in the WOS but not outside the WOS). The SNP may disrupt the binding site of a transcription factor which may result in aberrant expression of the target gene, but the loop itself is operational only in a particular condition. It is also possible that the SNP effects the loop strength, for example by perturbing the binding site of an architectural protein, e.g. CTCF, which may affect the regulation of a gene (de Wit et al., 2015; Tang et al., 2015; Guo et al., 2018). The TCE-*Pappa* interaction was conserved in human and rat. Therefore, we asked if we could examine additional variants associated with breast cancer for their participation in condition-specific long-range interactions (Figure 5).

We considered two studies for this problem, one that mapped SNPs to genes using a computational tool, INQUISIT (Zhang et al., 2020), and two, that used Capture-Hi-C (Baxter et al., 2018). We obtained 26 SNPs from a recent human breast cancer GWAS study that were linked to potential target genes in 201 interactions using INQUISIT (Zhang et al., 2020). We mapped these SNPs to loci in rn6 using liftOver and then identified target genes with the DCIs (Methods), (Kuhn et al., 2013). Since the lifted over position in rat likely does not correspond to a SNP in rat, we refer to the interactions as SNP-gene interactions in human and locus-gene interactions in rat. A total of 11 SNPs mapped to a locus in rat corresponding to a total of 101 human SNP-gene associations. Of these 11 SNPs, we identified 15 locus-gene DCIs, connecting 6 SNPs and 9 genes in total across these interactions (Zhang et al., 2020) (Table 3). Of these interactions, 7 locus-gene DCIs were within WOS (5 SNPs, 5 genes) and 8 locus-gene DCIs were outside WOS (4 SNPs, 6 genes). Of the 5 genes connected to within WOS locus-gene interactions, *Jag1* is differentially up-regulated within WOS (Figure 5A). *Jag1* is part of the notch signaling pathway involved in the renewal of stem and progenitor cells in mammary glands and has been associated with poor overall survival in breast cancer (Reedijk et al., 2005). Of the 6 genes connected to outside WOS locus-gene interactions, *Trps1* and *Phf1* are differentially up-regulated outside the WOS. Knockdown of *Trps1* results in reduced tumor growth in shRNA screens and *Trps1* has been shown to repress transcription by interacting with multiple components of the nucleosome remodeling deacetylase complex (Witwicki et al., 2018). *Phf1* is part of the polycomb group of proteins that maintain repressive chromatin states and has been shown to be an activator of the p53 signaling pathway (Figure 5B) (Yang et al., 2013). p53 is a tumor suppressor that regulates cell growth and apoptosis (Yang et al., 2013). Both *Trps1* and *Phf1* have tumor suppressor properties, are associated with repressive chromatin and are up-regulated outside WOS.

**TABLE 3:** Rat loci-gene interactions that recapitulate human breast cancer GWAS SNP-gene interaction from Zhang et al. (2020). Shown is the name of the human SNP, the rat 10 kb bin that has the gene and the variant of the conserved loop. The human SNP is named to show the chromosome ID and the genomic coordinate.

Gene name	Human SNP	Chr	GeneContainingBin	SNPContainingBin	Within or outside WOS
*Hnf1a*	rs17215231	12	47430000	47420000	Within
*Trps1*	rs13277568	7	90150000	90310000	Within
*Jag1*	rs141526427	3	130090000	131090000	Within
*Jag1*	rs141526427	3	130100000	131090000	Within
*Jag1*	rs141526427	3	130110000	131090000	Within
*Lekr1*	rs34052812	2	157570000	157440000	Within
*Mrps35*	chr12_29140260	4	181430000	182340000	Within
*Phf1*	rs2464195	20	5530000	5440000	Outside
*Cuta*	rs2464195	20	5530000	5440000	Outside
*Sppl3*	rs17215231	12	47310000	47420000	Outside
*Hnf1a*	rs17215231	12	47410000	47420000	Outside
*Trps1*	rs13277568	7	90190000	90310000	Outside
*Trps1*	rs13277568	7	90200000	90310000	Outside
*Trps1*	rs13277568	7	90300000	90310000	Outside
*Tiparp*	rs34052812	2	157340000	157440000	Outside

We performed a similar analysis by leveraging a study that used Capture Hi-C to link breast cancer GWAS SNPs to genes (Baxter et al., 2018). The study investigated 41 breast cancer GWAS SNPs connected to genes. Of these 41, 16 mapped to a locus in rat and participated in 63 SNP-gene interactions in the human Capture Hi-C data. Seven of these SNP-gene interactions mapped to a corresponding locus-gene DCI in rat, one in a within WOS DCI and six in outside WOS DCIs (5 SNPs, 6 genes, Table 4). For the within WOS DCI, the locus corresponded to human SNP rs13387042 and gene *Igfbp5* (Figure 5C). *Igfbp5*, like the WOS-associated gene *Pappa* gene, is involved in IGF signaling and plays an important role in mammary development (Wyszynski et al., 2016). The interaction between rs13387042 and *Igfbp5* is supported by previous studies in humans (Ghoussaini et al., 2014; Baxter et al., 2018). This result suggests that the mechanism by which variants interact with the *Igfbp5* promoter may be related to WOS.

**TABLE 4:** Rat loci-gene interactions that recapitulate human breast cancer GWAS SNP-gene interaction from Baxter et al. (2018), identified with Capture-Hi-C. Shown is the name of the human SNP, the rat 10 kb bin that has the gene and the variant of the conserved loop.

Gene name	Human SNP	Chr	GeneContainingBin	SNPContainingBin	Within or outside WOS
*Igfbp5*	rs13387042	9	80160000	80440000	Within
*Zmiz1*	rs704010	16	1890000	1760000	Outside
*Zmiz1*	rs704010	16	1930000	1760000	Outside
*Gtpbp3*	rs8170	16	19900000	19790000	Outside
*Rpl37a*	rs16857609	9	80000000	80810000	Outside
*Olfml3, Hipk1*	rs11552449	2	206220000	206290000	Outside
*Ovol1*	rs3903072	1	220930000	220910000	Outside

Of the 6 genes that are interacting with SNP-associated loci outside WOS, two are differentially expressed, *Ovol1* (up-regulated outside WOS) and *Olfml3* (up-regulated inside WOS). *Ovol1* has been shown to induce mesenchymal to epithelial transition in human cancers (Roca et al., 2013) and is associated with rs3903072. This association is also supported by human eQTL studies in breast cancer, suggesting that rs3903072 may alter *Ovol1* expression (Li et al., 2014). Targeting *Olfml3* has been shown to suppress tumor growth and angiogenesis (Stalin et al., 2021). Of the non-DE genes, *Zmiz1* is a prognostic marker of multiple cancer types (Mathios et al., 2019), *Rpl37a* is a biomarker for response to neoadjuvant chemotherapy in non-metastatic locally advanced breast cancer (Carrara et al., 2021), and *Hipk1* has been shown to act as a tumor suppressor by activating p53 (Rey et al., 2013). In the outside WOS DCI locus-gene interactions, *Hipk1* interacts with rs11552449. This interaction is also supported in human follicular helper T cell Capture Hi-C data (Su et al., 2020). Infiltration of follicular helper T cells has also been shown to predict breast cancer survival (Gu-Trantien et al., 2013). Overall, we were able to recover several human SNP locus-gene interactions in our dataset, which connected to genes implicated in cancer. The conservation of these long-range interactions in human and rat enable leveraging our dataset to study the human loci in rat as a model system.

## Discussion

The window of susceptibility (WOS) of breast cancer is an important period during which cancer risk due to environmental exposure is higher in women. The three-dimensional organization of the genome likely plays an important role in the transcriptional programs underlying the early stages of carcinogenesis (Henning et al., 2016; García-Nieto et al., 2017). However, little is known about these mechanisms within the WOS and how it differs outside the WOS. Here, we generated unique Hi-C and RNA-seq datasets for rats in and outside WOS and developed a computational approach, NE-MVNMF, that can unravel these differences.

Dynamics in three-dimensional genome organization can be studied at the level of individual loops as well as higher-order organizational units. However, the immediate impact on downstream gene expression due to these changes remains debated (van Steensel and Furlong, 2019). We demonstrated that differential chromatin interactions (DCIs) are associated with transcriptional differences between within WOS and outside WOS. Upregulated genes associated with differential interactions, which are higher in strength within WOS are specifically enriched for cell-cycle related terms compared to all up-regulated genes or genes associated with DCIs with counts higher outside the WOS. The cell cycle has been implicated in breast cancer susceptibility (Deng, 2006) and is often deregulated in breast cancer (Bower et al., 2017). Our results suggests that long-range regulation or deregulation of cell cycle genes could be important avenues for functional studies of breast cancer susceptibility.

A significant challenge in studying dynamics in 3D genome organization is detecting reliable changes between time points. This is difficult because of high sparsity of the data. To address this challenge, we developed a multi-view NMF approach with network enhancement to first enhance the Hi-C signal followed by identification of large-scale topological changes within WOS and outside WOS. The network enhancement smooths the matrix, which strengthens well-supported interactions and weakens poorly supported interactions. This allows MV-NMF to be more robust to noise and bias. Our results show that network enhancement to smooth the matrices before NMF leads to the identification of dynamic blocks that have larger changes in contact count overall and specifically larger changes in DCIs compared to static blocks. Closer inspection of dynamic blocks revealed many genes that are involved in mammary development and cancer-associated pathways.

We previously identified a WOS-specific interaction between the TCE and the *Pappa* gene (Henning et al., 2016). This interaction is conserved across human, rat and mouse. Therefore, we asked if we can identify similar conserved interactions by mapping human SNP-gene interactions to rat, which can then be followed up with in-depth molecular characterization in a model organism. We identified several examples of conserved locus-gene interactions by comparing our DCIs to two previous studies connecting breast cancer susceptibility SNPs to genes in human (Baxter et al., 2018; Zhang et al., 2020). For example, the SNP rs13387042, which falls in an enhancer region in human loops over a distance of 400 kb to *Igfbp5* (Wyszynski et al., 2016). We were able to map this locus onto a DCI within WOS rats connected to the rat ortholog of *Igfbp5.* Notably, similar to the previously validated WOS-associated *Pappa* gene, *Igfbp5* is also involved in mammary development and IGF signaling. This interaction, along with the other interactions identified in this study, will be a valuable resource for enabling deeper characterization of genetic variation and breast cancer that may have a similar age-specific window of susceptibility.

Our work can be extended in several ways. First, the addition of more time points would be useful in identifying more fine-grained dynamics of chromatin for entry and exit from the WOS. Second, the addition of one-dimensional regulatory signals would be beneficial in determining which enhancers and promoters are active within and outside WOS. In general, a more robust dataset can aid in gaining a more complete picture of the molecular mechanisms underlying WOS. On the methodological side, our approach could be extended to identify more complex patterns of change in 3D genome organization to handle more time points and heterogeneous samples. Taken together, our transcriptomic and 3D genome profiles of within WOS and outside WOS and our computational pipeline should be a useful resource for studying the role of 3D genome organization in the window of susceptibility for breast cancer.

## Data Availability

The datasets generated for this study can be found in the GEO database with the accession number GSE184285 (https://www.ncbi.nlm.nih.gov/geo/query/acc.cgi?acc=GSE184285). Source code (C++) for MVNMF is available here: http://github.com/Roy-lab/mvnmf.
