# Supplementary material for: Deciphering the Role of 3D Genome Organization in Breast Cancer Susceptibility
Source: Front Genet. 2022 Jan 11;12:788318. doi: 10.3389/fgene.2021.788318 (PMC8787344; doi:10.3389/fgene.2021.788318)
Supplement: Supplementary file 1 [file DataSheet1.docx]

Supplementary Material

# Supp Figures and Tables

## Supp Figures


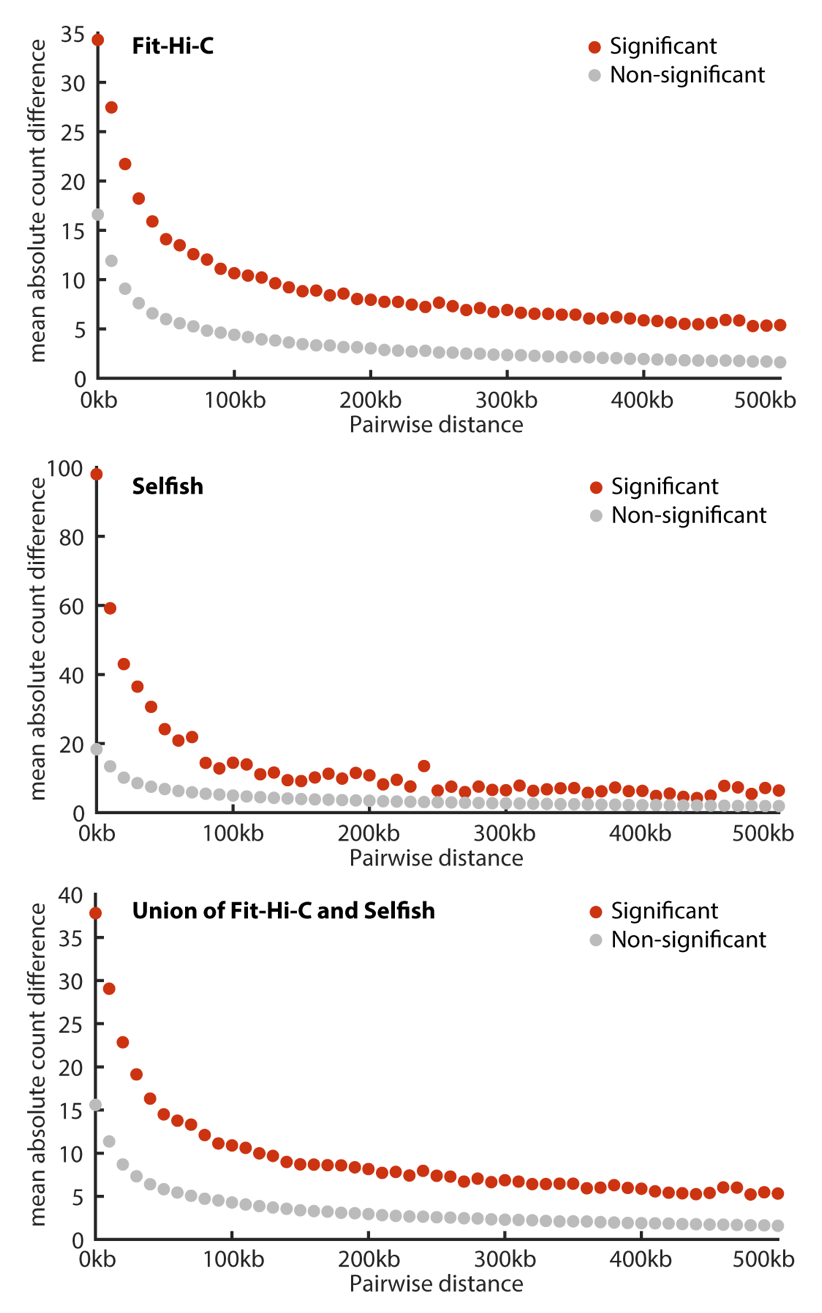


**Supp Figure 1.** Mean absolute interaction count difference between within-WOS and outside-WOS DCI. DCIs were identified using Fit-Hi-C (top), Selfish (middle), and by taking the union of DCIs identified by both methods (bottom).


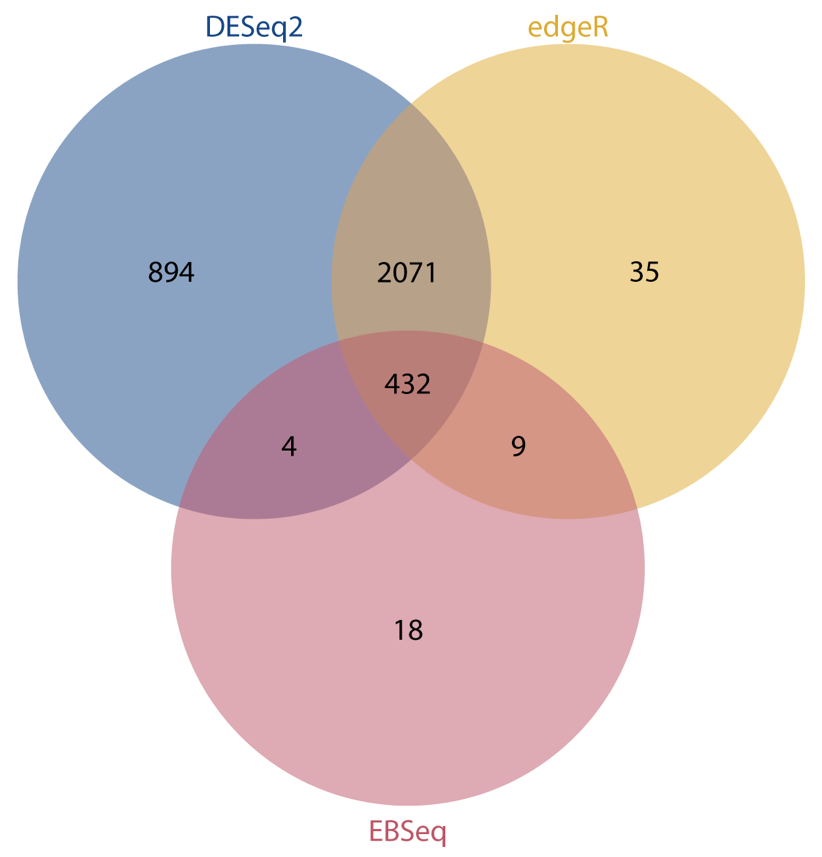


**Supp Figure 2.** Number of DE genes identified by three different methods (EBSeq, DESeq2 and edgeR) and the size of their intersections.


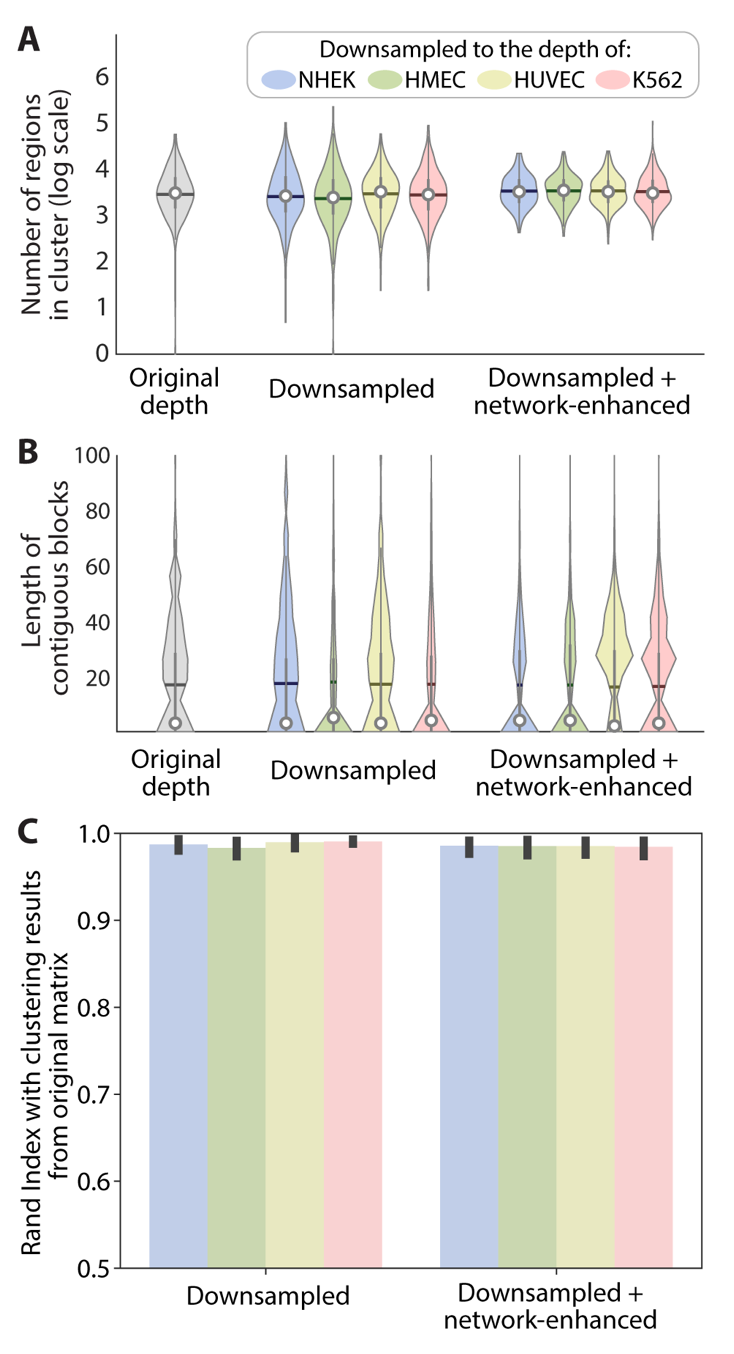


**Supp Figure 3.** The effect of network enhancement (NE) on NMF cluster size, contiguous block length, and similarity of clustering results in sparser, downsampled input data. **A.** The distribution of NMF cluster sizes from original, high-depth GM12878 cell line (grey), and those from input data downsampled to lower-depth cell lines: NHEK (blue), HMEC (green), HUVEC (yellow), K562 (pink), with and without NE. **B.** The distribution of contiguous block lengths from high-depth GM12878 cell line (grey), and those from input data downsampled to lower-depth cell lines: NHEK (blue), HMEC (green), HUVEC (yellow), K562 (pink), with and without NE. **C.** Similarity between clustering results from high-depth input data (from GM12878 cell line) and those from inputs downsampled to various depths (NHEK – blue, HMEC – green, HUVEC, yellow, K562 – pink), with and without NE.


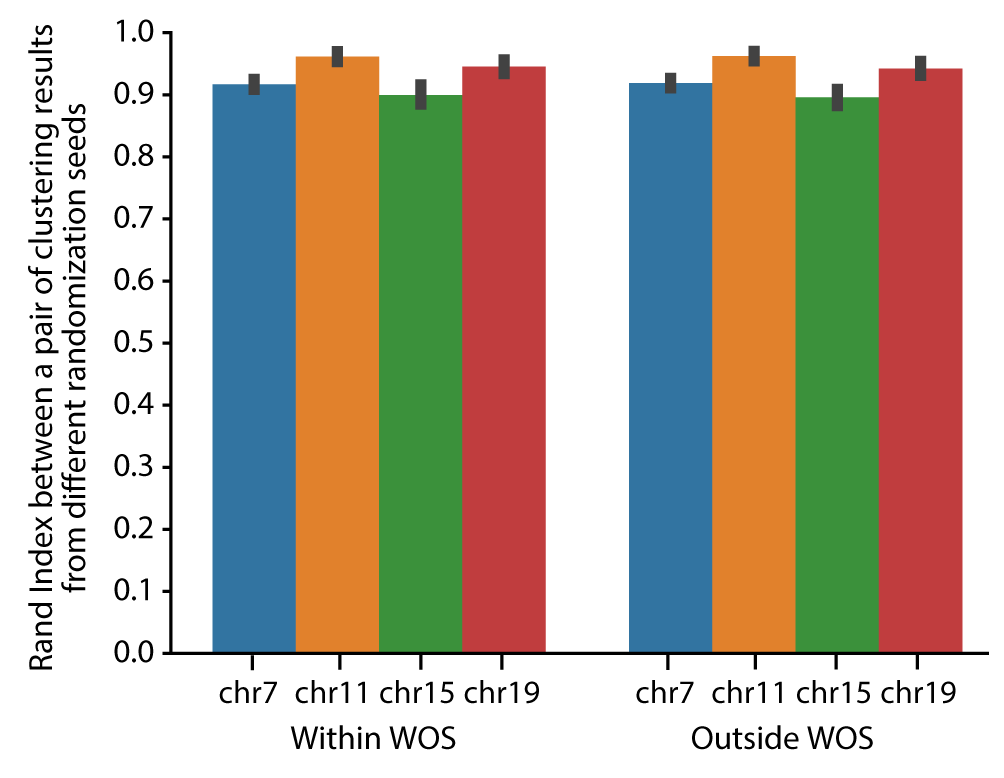


**Supp Figure 4.** Stability of the MVNMF clustering results to random initialization, measured by Rand Index between a pair of clustering results from different random initialization seeds.

##
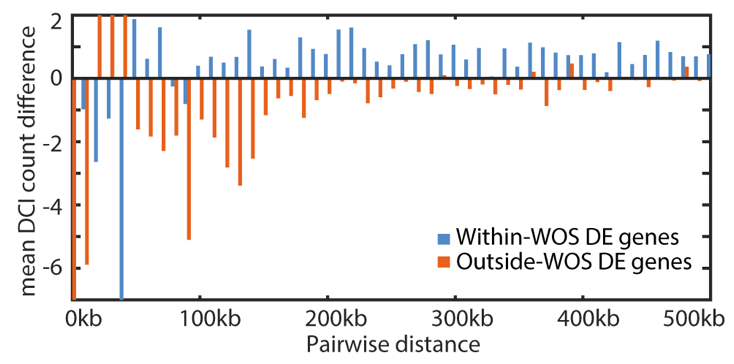


**Supp Figure 5.** Mean count difference (within-WOS count – outside-WOS count) for DCIs associated with genes that are up-regulated within WOS (i.e., within-WOS DE genes, blue) and up-regulated outside WOS (outside-WOS DE genes, orange). Genes were associated with any within-WOS or outside-WOS DCI, regardless of whether the gene expression itself is up-regulated within WOS or outside WOS.

**
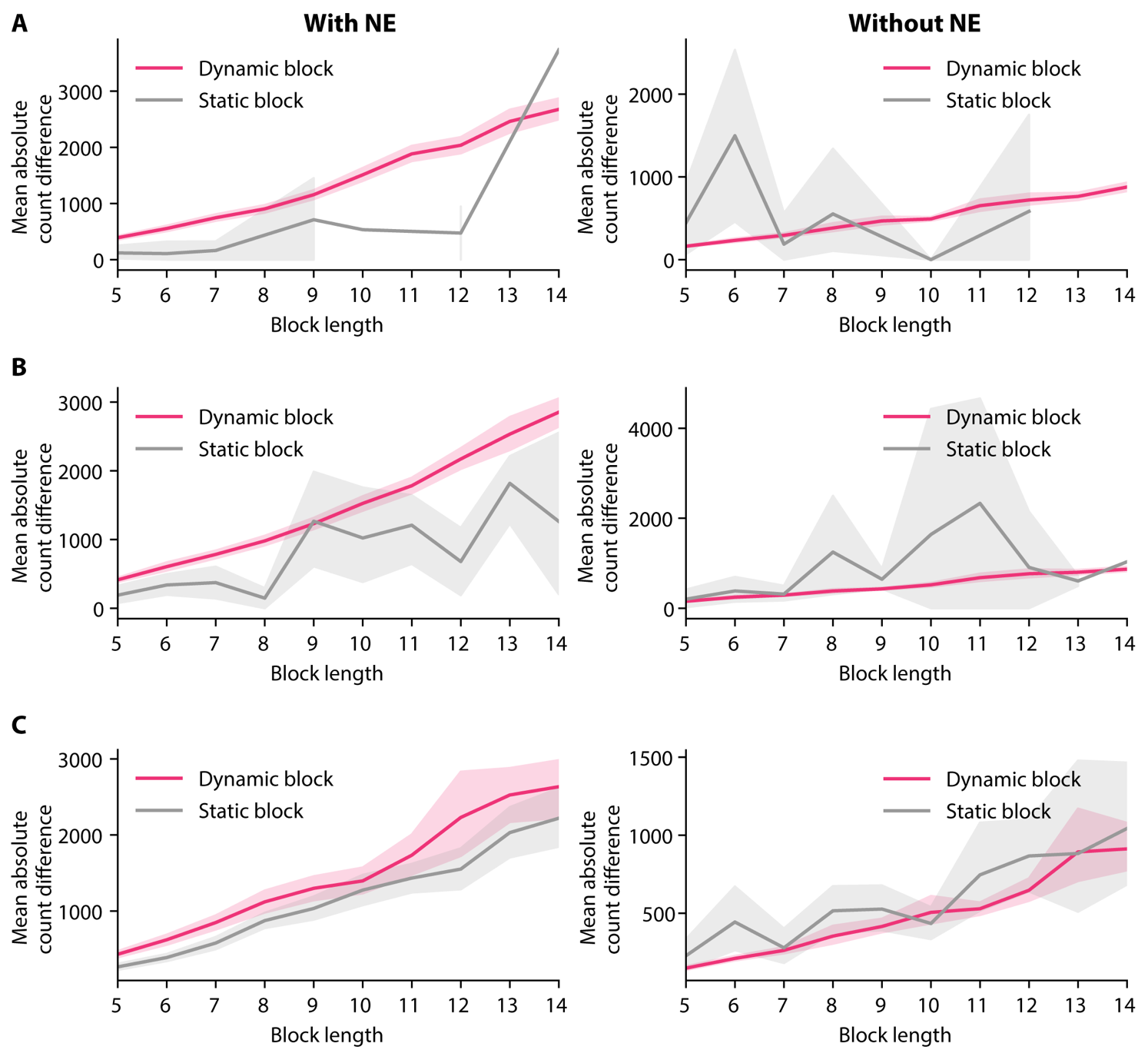
**

**Supp Figure 6.** Difference in interaction counts among regions within dynamic blocks and static blocks for different values of the hyperparameter α, which controls the strength of regularization that makes the factor matrices similar to the consensus factor. For each dynamic or static block, we summed up the absolute value difference between interactions from within WOS and those from outside WOS. We plot the mean of the absolute value difference by block size with NE (left panels) and without NE (right panels). The shaded area represents the 95% confidence interval. **A.** Mean absolute count difference under α = 10^5^. **B.** α = 10^6^. **C.** α = 10^7^. For α = 10^8^, see **Figure 3C**.

## Supplementary Tables

**Supplemental Table 1**. Number of dynamic and static blocks by alpha and network enhancement

|  | Num static blocks NE | Num dynamic blocks NE | Num static blocks no NE | Num dynamic blocks no NE |
| --- | --- | --- | --- | --- |
| 1e-5 | 66 | 4894 | 20 | 4998 |
| 1e-6 | 229 | 4641 | 82 | 4392 |
| 1e-7 | 1745 | 506 | 585 | 1097 |
| 1e-8 | 1930 | 168 | 480 | 794 |
